# IPSS-M has greater survival predictive accuracy compared with IPSS-R in persons ≥ 60 years with myelodysplastic syndromes

**DOI:** 10.1186/s40164-022-00328-4

**Published:** 2022-10-17

**Authors:** Junying Wu, Yudi Zhang, Tiejun Qin, Zefeng Xu, Shiqiang Qu, Lijuan Pan, Bing Li, Yujiao Jia, Chengwen Li, Huijun Wang, Qingyan Gao, Wenyu Cai, Jingye Gong, Songyang Zhao, Fuhui Li, Robert Peter Gale, Zhijian Xiao

**Affiliations:** 1 State Key Laboratory of Experimental Hematology, National Clinical Research Center for Blood Diseases, Haihe Laboratory of Cell Ecosystem, Institute of Hematology & Blood Diseases Hospital, Chinese Academy of Medical Sciences & Peking Union Medical College, Tianjin, 300020 China; 2 MDS and MPN Centre, Institute of Hematology & Blood Diseases Hospital, Chinese Academy of Medical Sciences & Peking Union Medical College, Tianjin, 300020 China; 3 Hematologic Pathology Centre, Institute of Hematology & Blood Diseases Hospital, Chinese Academy of Medical Sciences & Peking Union Medical College, Tianjin, 300020 China; 4 Centre for Hematology, Department of Immunology and Inflammation, Imperial College of Science, Technology and Medicine, London, UK

**Keywords:** Myelodysplastic syndrome, Prognostic model, Patient age, Mutation profile

## Abstract

There are considerable new data on mutation topography in persons with myelodysplastic syndromes (MDS). These data have been used to update conventional risk models such as the Revised International Prognostic Scoring System (IPSS-R). Whether the molecular IPSS (IPSS-M) which includes these data improves survival prediction accuracy is untested. To answer this question, we compared survival prediction accuracies of the IPSS-R and IPSS-M in 852 consecutive subjects with de novo MDS. Concordance statistics (C-statistics) of the IPSS-R and IPSS-M in the entire cohort were similar, 0.67 (95% Confidence Interval [CI] 0.64, 0.71) and 0.68 (0.64, 0.71). Average numbers of mutations and of IPSS-M related mutations were greater in persons ≥ 60 years (2.0 [Interquartile Range [IQR], 1, 3] *vs.* 1.6 [0, 2], *P* = 0.003; 1.6 [0, 2] *vs.* 1.3 [0, 2], *P* = 0.006). Subjects ≥ 60 years had a higher incidence of mutations in *RUNX1, TP53*, *TET2, SRSF2, DNMT3A, STAG2*, *EZH2* and *DDX41*. In contrast, mutations in *U2AF1* were more common in persons < 60 years. Next we tested survival prediction accuracy based on age < or ≥ 60 years. C-statistics of the IPSS-R and IPSS-M in subjects ≥ 60 years were 0.66 (0.61, 0.71) and 0.69 (0.64, 0.73) whereas in subjects < 60 years they were 0.67 (0.61, 0.72) and 0.65 (0.59, 0.71). These data indicate an advantage for the IPSS-M over the IPSS-R in subjects ≥ 60 years but not in those < 60 years probably because of a great frequency of mutations correlated with survival in those ≥ 60 years.

**Supplementary Information:**

The online version contains supplementary material available at 10.1186/s40164-022-00328-4.

## Background

Myelodysplastic syndromes (MDS) are heterogeneous cancers. Accurate survival prediction models are important in counseling persons with MDS and choosing therapy(ies). The International Prognostic Scoring System (IPSS) and revised version of IPSS (IPSS-R) are the most commonly used employing hematological, histological and cytogenetic data for survival estimation [1, 2]. Recently, considerable data on the mutation topography of persons with MDS have become available and have been added to the aforementioned co-variates to predict survival [3–9]. An example is the molecular IPSS (IPSS-M) which adds data on 31 mutations to classify people with MDS into six survival strata [6]. Because the mutation topographies of persons < and ≥ 60 years differ, we wondered whether the IPSS-M was more accurate compared with the IPSS-R in different age cohorts. Interrogating data from 852 consecutive subjects we found an advantage for the IPSS-M in subjects ≥ 60 years but not in those < 60 years.

## Methods

### Subjects

We interrogated data from 852 consecutive subjects with newly-diagnosed de novo MDS seen at our centre from August, 2016 to September, 2021. Diagnosis was based on the 2016 revised criteria of the World Health Organization (WHO). 760 (89%) subjects had evaluable karyotypes at diagnosis which were classified according to the IPSS-R criteria. IPSS-R and IPSS-M model risk scores were calculated. Follow-up data were available in 771 subjects (91%). Median follow-up of survivors was 25 months (Interquartile Range [IQR], 8, 31 months). Overall survival (OS) was defined as the duration from the date of diagnosis to date of death or last follow-up. For patients who received Allo-HSCT, OS was calculated from the data of diagnosis to the date of transplant. A total of 550 subjects (65%) of the 852 MDS subjects were male. Median age was 56 years (IQR: 44, 64 years) and 540 subjects (63%) were < 60 years old. Co-variates of the subjects were displayed in Additional file 1: Table S1. Treatment data were available in 707 subjects (83%) 0.40 subjects (5.7%) accepted erythropoietin with or without G-CSF, red blood cell and/or platelet transfusions. 316 patients (44.6%) received immunosuppressive therapy (cyclosporine, thalidomide and danazol). 160 subjects (18.8%) accepted decitabine or azacytidine and 31 (3.8%) accepted chemotherapy including aclacinomycin or homoharringtonine combined with cytarabine and granulocyte-colony stimulating factor (G-CSF; termed CAG or HAG), idarubicin or daunorubicin combined with cytarabine (IA or DA) or melphalan. A total of 111 patients (13%) received Allo-HSCT. And some other patients (49 subjects; 5.8%) accepted traditional Chinese medicines. The study was approved by the Ethical Committee on Medical Research at Institute of Hematology and Blood Disease Hospital, conducted in accordance with the principles of the Declaration of Helsinki and all subjects gave written informed consent.

### Targeted gene sequencing

Mutation sequencing was done in 592 subjects using a 141-gene panel from August, 2016 to March, 2020 (Additional file 1: Table S2) and in 260 subjects using a targeted gene sequencing of 267 genes from April, 2020 to September 2021 at diagnosis in our center (Additional file 1: Table S3 and S4). *PRPF8* and *GNB1*, defined as residual genes in IPSS-M model, were not included in the 141-gene panel. Functionally annotated mutations were filtered by 1000 Genomes, ESP6500, Inhouse, PolyPhen, SIFT and COSMIC to determine pathogenicity as described [10]. *TP53* allelic state was determined as described [11, 12]. We grouped mutations into 16 main effect genes (*ASXL1, CBL, DNMT3A, ETV6, EZH2, FLT3, IDH2, KRAS, MLL*^*PTD*^*, NPM1, NRAS, RUNX1, SF3B1, SRSF2, TP53*^*multihit*^ and *U2AF1*) and 15 residual genes (*BCOR, BCORL1, CEBPA, ETNK1, GATA2, GNB1, IDH1, NF1, PHF6, PPM1D, PRPF8, PTPN11, SETBP1, STAG2* and *WT1*) as in the IPSS-M model [6]. Details of targeted gene sequencing are in the Additional file 1.

### Statistics

Numerical variables between groups were tested by the Mann–Whitney U or Kruskal Wallis tests. Categorical co-variates were compared with the Fisher exact or the *χ*^2^ tests. Survival was defined as the interval from diagnosis to death or last follow-up. The OS was estimated using the Kaplan–Meier method and compared by the log-rank test in univariable analyses. Multi-variable survival analysis used a Cox proportional-hazards regression model which included co-variates significant in uni-variable analyses at *P* < 0.20. Two-sided *P* values < 0.05 were considered of statistical significance. Model prediction accuracies were assessed by time-depend area under receiver-operator characteristic (AUROC) curves expressed as a Concordance (C)-statistic [13]. Data were analysed using SPSS version 25.0 (IBM, Chicago, IL, USA) and R statistical language (R Development Core 2008). Additional information is in the Additional file 1.

## Results

### Mutation topography

**Fig. 1:**
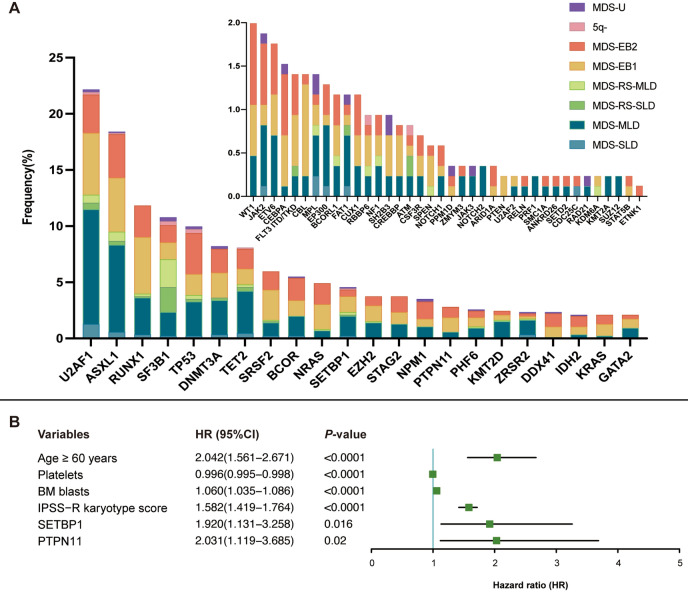
Genomic landscape and multivariable survival analysis in 852 patients with de novo myelodysplastic syndromes. **A** The frequency of mutated genes in 852 patients with de novo MDS. Lesions observed in more than five patients are shown. Colors represent different 2016 WHO subtypes. **B** Significant variables in multivariable analysis in 852 subjects with de novo MDS. Abbreviations: MDS: myelodysplastic syndrome; MDS-U: unclassified MDS; SLD: single-lineage dysplasia; MLD: multilineage dysplasia; RS: ring sideroblasts; EB: excess blasts; WHO: World Health Organization; BM: bone marrow; IPSS-R: International Prognostic Scoring System-Revised

We characterized the genomic landscape of our MDS subjects. 652 subjects (77%) had ≥ 1 relevant mutation, 404 (47%) had ≥ 2 and 228 (27%) ≥ 3. Median number of mutations was 1.74 (IQR, 1–3). A total of 750 (88%) patients presented at least one pathogenic molecular abnormality. 290 subjects (34%) having mutations only, 90 (11%) abnormal cytogenetics only and 297 (35%) both. The five most frequent mutations included *U2AF1* (22%), *ASXL1* (18%), *RUNX1* (12%), *SF3B1* (11%) and *TP53* (10%; Fig. 1A).

### Survival

In univariable analysis, age ≥ 60 years old, hemoglobin levels, platelet concentrations, BM blast percentage, IPSS-R cytogenetic scores and mutations in *RUNX1, SF3B1, TP53, SRSF2, PTPN11, CEBPA* were identified as significant factors for prognosis (*P* < 0.05, Table 1). In multi-variable analyses, age ≥ 60 years old (Hazard Ratio [HR] = 2.04; 95% Confidence Interval [CI], 1.56, 2.67; *P* < 0.0001), platelet concentrations (HR = 1.0 [1.0, 1.0]; *P* < 0.0001), bone marrow blasts (HR = 1.06 [1.04, 1.09]; *P* < 0.0001), IPSS-R cytogenetic scores (HR = 1.58 [1.42, 1.76]; *P* < 0.0001), *SETBP1* mutation (HR = 1.92 [1.13, 3.26]; *P* = 0.016) and *PTPN11* mutation (HR = 2.03 [1.12, 3.69]; *P* = 0.02) correlated with survival (Table 1; Fig. 1B).

**Table 1:** Multivariable analysis of prognostic factors for overall survival in patients with MDS

Variables	Univariable	*P*	Multivariable	*P*
	HR (95% CI)		HR (95% CI)	
Age ≥ 60 years	1.037 (1.027–1.048)	** < 0.0001**	2.042 (1.561–2.671)	** < 0.0001**
Hemoglobin	0.994 (0.989–1.000)	**0.037**		
ANC	0.996 (0.927–1.071)	0.920		
Platelets	0.997 (0.995–0.998)	** < 0.0001**	0.996 (0.995–0.998)	** < 0.0001**
BM blasts	1.084 (1.061–1.107)	** < 0.0001**	1.060 (1.035–1.086)	** < 0.0001**
IPSS-R karyotype score	1.581 (1.414–1.768)	** < 0.0001**	1.582 (1.419–1.764)	** < 0.0001**
*U2AF1*	1.145 (0.855–1.533)	0.365		
*ASXL1*	1.178 (0.872–2.047)	0.286		
*RUNX1*	1.438 (1.011–0.989)	**0.040**		
*SF3B1*	0.618 (0.396–0.965)	**0.031**		
*TP53*	2.823 (2.024–3.938)	** < 0.0001**		
*DNMT3A*	1.439 (0.966–2.143)	0.074		
*TET2*	1.400 (0.919–2.131)	0.117		
*SRSF2*	1.589 (1.036–2.438)	**0.034**		
*BCOR*	0.837 (0.457–1.530)	0.563		
*NRAS*	1.628 (0.981–2.699)	0.059		
*SETBP1*	1.528 (0.922–2.534)	0.094	1.920 (1.131–3.258)	**0.016**
*EZH2*	1.647 (0.960–2.824)	0.065		
*STAG2*	1.066 (0.566–2.007)	0.808		
*NPM1*	1.090 (0.514–2.309)	0.823		
*PTPN11*	2.031 (1.137–3.626)	**0.014**	2.031 (1.119–3.685)	**0.020**
*PHF6*	1.516 (0.806–2.853)	0.190		
*KMT2D*	0.637 (0.263–1.544)	0.310		
*ZRSR2*	0.882 (0.364–2.138)	0.779		
*DDX41*	1.387 (0.712–2.700)	0.329		
*IDH2*	1.867 (0.922–3.780)	0.075		
*KRAS*	1.472 (0.654–3.311)	0.342		
*GATA2*	0.941 (0.419–2.115)	0.887		
*WT1*	1.044 (0.389–2.804)	0.932		
*JAK2*	0.538 (0.172–1.680)	0.274		
*ETV6*	1.070 (0.342–3.343)	0.908		
*CEBPA*	2.616 (1.163–5.884)	**0.015**		
*FLT3 ITD/TKD*	0.651 (0.162–2.620)	0.546		
*CBL*	2.156 (0.958–4.853)	0.055		
*MPL*	1.041 (0.334–3.251)	0.944		
*BCORL1*	0.049 (0.00–19.796)	0.325		
*FAT1*	0.753 (0.187–3.030)	0.685		
*CUX1*	0.359 (0.050–2.559)	0.306		

*P* < 0.05 were indicated in Bold

*HR* hazard ratio, *CI* confidence interval, *ITD* internal tandem duplication, *TKD* tyrosine kinase domain

### Re-classification from IPSS-R to IPSS-M

**Fig. 2:**
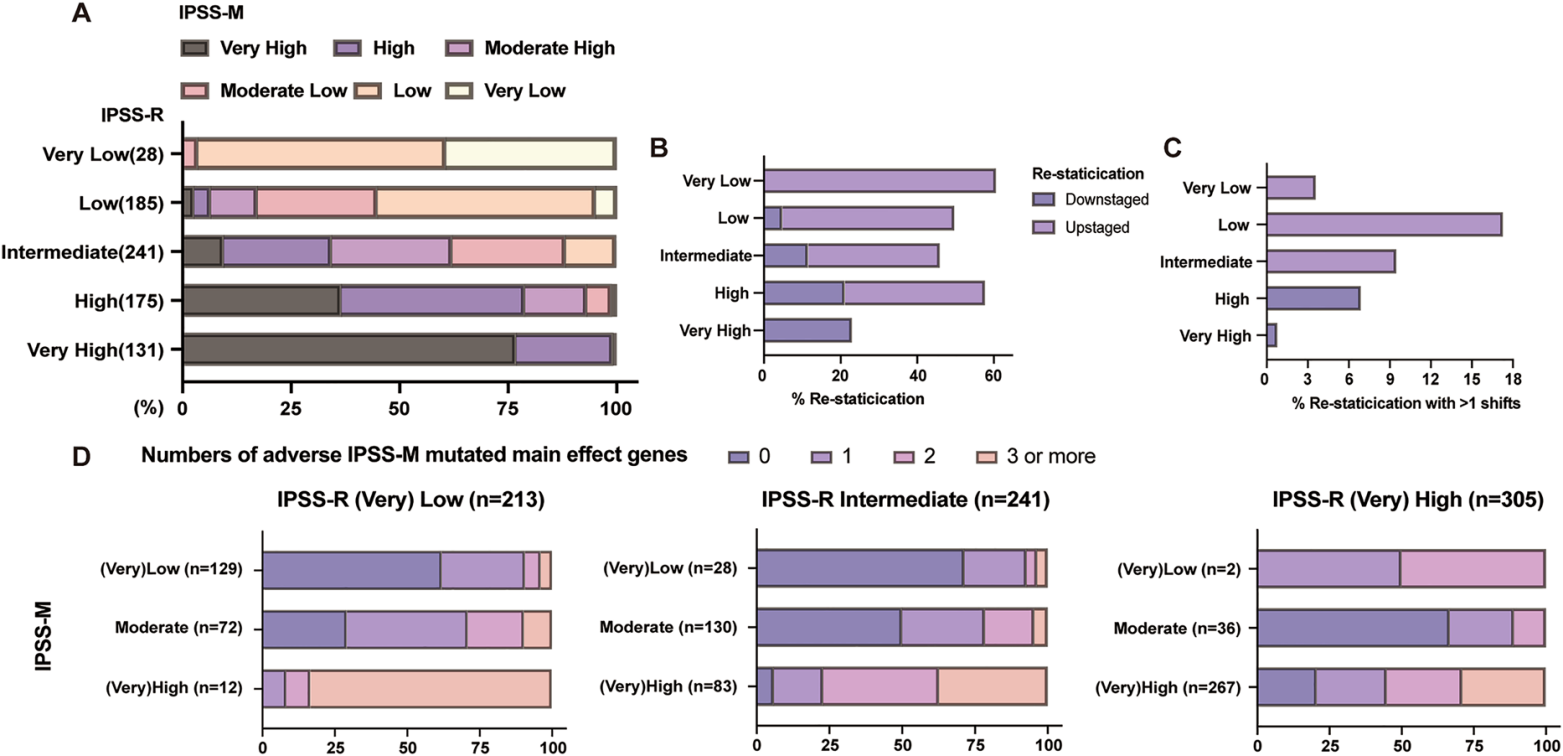
Comparison of the International Prognostic Scoring System-Revised (IPSS-R) and the International Prognostic Scoring System-Molecular (IPSS-M). **A** The restratification of IPSS-R to IPSS-M for 852 patients with MDS. Vertical axis represents IPSS-R categories and horizontal axis represents IPSS-M categories. The proportion of patients in each category is shown in Additional file 1: Table S6. **B**, **C** The percentage of restratified patients in each IPSS-R stratum, counting either any shift or cases with more than one shifts. **D** The association between the number of mutated IPSS-M main effect adverse genes and patient reclassification. Abbreviations: MDS: myelodysplastic syndrome; BM: bone marrow; IPSS-R: International Prognostic Scoring System-Revised; IPSS-M: International Prognostic Scoring System-Molecular

Subjects were classified using the IPSS-R and IPSS-M models (Additional file 1: Table S6 and S7). When analyzing the restratification of patients from IPSS-R to IPSS-M (by merging moderate low and moderate high into moderate in IPSS-M), 351 subjects (41%) were re-classified. Of these subjects, 247 (70%) were up-staged and 104 (30%), down-staged (Additional file 1: Table S7; Fig. 2). 83 (45%) patients in the IPSS-R low risk category were up-staged into higher-risk categories (moderate/high/very high) in the IPSS-M. In IPSS-R intermediate group, 12% patients were shifted into IPSS-M low risk category and 34% were reclassified as high/very high risk categories in IPSS-M model. 12% patients of the IPSS-R high/very high risk categories were down-staged into lower risk group in IPSS-M (Additional file 1: Table S7; Fig. 2). 144 of 159 subjects (91%) re-classified as very low/low IPSS-M cohorts had ≤ 1 IPSS-M mutation. 60 of 95 subjects (63%) re-classified from very low/low/intermediate in IPSS-R to very high/high IPSS-M had > 2 IPSS-M mutations (Fig. 2).

The IPSS-M could better stratify patients within the IPSS-R scoring system, the median OS of intermediate IPSS-R patients reclassified as moderate, high and very high IPSS-M was not reached, 34 months and 13 months (95% CI 8 to 18 months, *P* = 0.025; Additional file 1: Fig. S1). However, the IPSS-R did not classify patient outcomes in each IPSS-M risk group (Additional file 1: Fig. S1).

### Prognostic accuracy of IPSS-R and IPSS-M

**Fig. 3:**
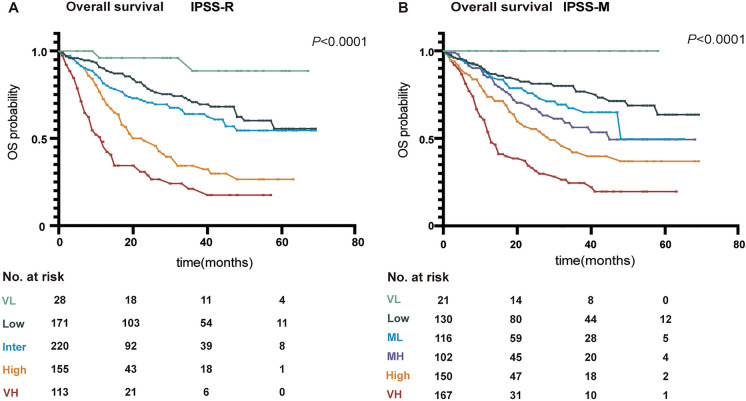
Overall survival of MDS patients stratified according to different prognostic scoring systems. Kaplan–Meier representation of each scoring systems in our cohort. **A** IPSS-R risk categories, **B** IPSS-M risk categories. *P-*values are from the log-rank test. Abbreviations: IPSS-R: International Prognostic Scoring System-Revised; IPSS-M: International Prognostic Scoring System-Molecular; OS: overall survival; VL: very low; Inter: intermediate; VH: very high

**Fig. 4:**
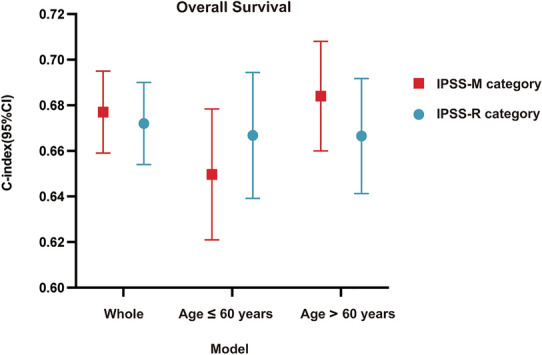
Comparison of prognostic model discrimination in patients with MDS. Model discrimination as measured by the C-statistics obtained with IPSS-R or IPSS-M categories on overall survival in the whole cohort, age < 60 years cohort and age ≥ 60 years cohort. Abbreviations: IPSS-R: International Prognostic Scoring System-Revised; IPSS-M: International Prognostic Scoring System-Molecular; CI: confidence interval

Using the IPSS-R model, 28 subjects (3%) were classified as very low risk, 185 (22%) as low-risk, 241 (28%) as intermediate-risk, 175 (21%) as high-risk and 131 (15%) as very high-risk. 3-year survivals were 92% (95% CI 0.78, 1.0), 72% (0.64, 0.80), 60% (0.52, 0.68), 39% (0.28, 0.5) and 20% (0.08, 0.31) (*P* < 0.0001: Fig. 3). Using the IPSS-M model, 21 subjects (3%) were classified as very low-risk, 138 (16%) as low-risk, 125 (15%) as moderate low-risk, 113 (13%) as moderate high-risk, 170 (20%) as high-risk and 192 (23%) as very high-risk. 3-year survivals were 100%, 80% (95% CI, 0.72, 0.88), 67% (0.57, 0.78), 56% (0.44, 0.69), 42% (0.31, 0.53) and 25% (0.15, 0.34) (*P* < 0.0001; Fig. 3). C-statistics for the IPSS-R and IPSS-M model were similar, 0.67 (95% CI 0.64, 0.71) and 0.68 (0.64, 0.71) (Fig. 4).

### Correlations between age and numbers and frequencies of mutations

In our dataset, survival was significantly longer in subjects < 60 years compared with those ≥ 60 years, not reached *versus* 25 months (19, 31 months; *P* < 0.0001; Additional file 1: Fig. S2).

Table 2 displays differences in clinical, hematologic and mutation data of subjects < and ≥ 60 years. Older subjects were more often male (72% *versus* 60%; *P* < 0.0001), more often had MDS with excess blasts (MDS-EB; 53% *versus* 36%; *P* < 0.0001), a higher percentage of bone marrow blasts (median: 4.5% *versus* 2.0%; *P* < 0.0001) and more often had IPSS-R poor-risk cytogenetics (14% *versus* 9%; *P* < 0.0001). Subjects < 60 years were more often had IPSS-R intermediate-risk cytogenetics (26% *versus* 15%; *P* < 0.0001). Although overall there was no significant difference in IPSS-R risk stratification based on age (*P* = 0.33), there were more IPSS-M subjects with very high-risk in subjects ≥ 60 years (28 *versus* 19%; *P* = 0.03, Table 2).

**Table 2:** Clinical, laboratory and genetic characteristics of younger (< 60 years) and older (≥ 60 years) MDS patients

Characteristic	Total (n = 852)	Age < 60 years (n = 540)	Age ≥ 60 years (n = 312)	*P-*value
Age (years)*	56 (44–64)	48 (37–55)	61 (63–70)	
Sex n (%)				< 0.0001
Male	550 (64.6)	325 (60.2)	225 (72.1)	
Female	302 (35.4)	215 (39.8)	87 (27.9)	
BM blasts (%)*	2.5 (17)	2 (0.5–6)	4.5 (1–8)	< 0.0001
(Missing)	1	1	0	
Hemoglobin (g/L)*	79 (66–95)	78 (65–96)	79 (66–95)	0.703
(Missing)	0	0	0	
Platelets (× 10^9^/L)*	60 (31–119)	59 (29–121)	63 (35–117)	0.260
(Missing)	0	0	0	
ANC (× 10^9^/L)*	1. (0.7–2)	1.1 (0.7–2)	1.1 (1.6–2)	0.930
(Missing)	0	0	0	
IPSS-R karyotype				< 0.0001
Very good	10 (1.2)	4 (0.7)	6 (1.9)	
Good	427 (50.1)	261 (48.3)	166 (53.2)	
Intermediate	186 (21.8)	138 (25.6)	48 (15.4)	
Poor	42 (4.9)	32 (5.9)	10 (3.2)	
Very poor	95 (11.2)	50 (9.3)	45 (14.4)	
(Missing)	92	55	37	
IPSS-R category				0.332
Very low	28 (3.3)	19 (3.5)	9 (2.9)	
Low	185 (21.7)	118 (21.9)	67 (21.5)	
Intermediate	241 (28.3)	165 (30.6)	76 (24.4)	
High	175 (20.5)	105 (19.4)	70 (22.4)	
Very high	131 (15.4)	78 (14.4)	53 (17.0)	
(Missing)	92	55	37	
IPSS-M category				0.027
Very low	21 (2.5)	16 (3.0)	5 (1.6)	
Low	138 (16.2)	92 (17.0)	46 (14.7)	
Moderate low	125 (14.7)	81 (15.0)	44 (14.1)	
Moderate high	113 (13.3)	82 (15.2)	31 (9.9)	
High	170 (20.0)	108 (20.0)	62 (19.9)	
Very high	192(22.5)	106(19.4)	87(27.9)	
(Missing)	93	55	37	
WHO 2016 subtypes				< 0.0001
MDS-SLD/MLD	414 (48.6)	295 (54.6)	119 (38.1)	
MDS-RS-SLD/MLD	46 (5.4)	24 (4.4)	22 (7.0)	
MDS-EB1/2	359 (42.1)	195 (36.1)	164 (52.5)	
5q- syndrome	12 (1.4)	8 (1.5)	4 (1.3)	
Unclassified MDS	21 (2.5)	18 (3.3)	3 (1.0)	
(Missing)	0	0	0	
Mutations				
*U2AF1*	189 (22.2)	141 (26.1)	48 (15.4)	< 0.0001
*ASXL1*	157 (18.4)	90 (16.7)	67 (21.5)	0.081
*RUNX1*	101 (11.9)	51 (9.4)	50 (16.0)	0.004
*SF3B1*	92 (10.8)	51 (9.4)	41 (13.1)	0.094
*TP53*	85 (10.0)	41 (7.6)	44 (14.1)	0.002
*DNMT3A*	70 (8.2)	36 (6.7)	34 (10.9)	0.030
*TET2*	69 (8.1)	32 (5.9)	37 (11.9)	0.002
*TP53*^*multihit*^	53 (6.2)	27 (5.0)	26 (8.3)	0.052
*SRSF2*	51 (6.0)	16 (3.0)	35 (11.2)	< 0.0001
*BCOR*	47 (5.5)	28 (5.2)	19 (6.1)	0.577
*NRAS*	42 (4.9)	26 (4.8)	16 (5.1)	0.839
*SETBP1*	39 (4.6)	25 (4.6)	14 (4.5)	0.924
*EZH2*	32 (3.8)	14 (2.6)	18 (5.8)	0.019
*STAG2*	32 (3.8)	10 (1.9)	22 (7.1)	< 0.0001
*NPM1*	30 (3.5)	24 (4.4)	6 (1.9)	0.054
*PTPN11*	24 (2.8)	13 (2.4)	11 (3.5)	0.342
*PHF6*	22 (2.6)	14 (2.6)	8 (2.6)	0.980
*KMT2D*	21 (2.5)	13 (2.4)	8 (2.6)	0.885
*ZRSR2*	20(2.3)	11(2.0)	9 (2.9)	0.432
*DDX41*	20(2.3)	5(0.9)	15 (4.8)	< 0.0001
*IDH2*	18(2.1)	10(1.9)	8 (2.6)	0.486
*KRAS*	18(2.1)	12(2.2)	6 (1.9)	0.770
*GATA2*	18(2.1)	13(2.4)	5 (1.6)	0.431
*WT1*	17(2.0)	14(2.6)	3 (1.0)	0.101
*JAK2*	16(1.9)	9(1.7)	7 (2.2)	0.550
*ETV6*	15(1.8)	11(2.0)	4 (1.3)	0.420
*CEBPA*	13 (1.5)	7 (1.3)	6 (1.9)	0.472
*FLT3 ITD/TKD*	14 (1.6)	8 (1.5)	6 (1.9)	0.625
*CBL*	12 (1.4)	7 (1.3)	5 (1.6)	0.715
*MPL*	12 (1.4)	10 (1.9)	2 (0.6)	0.148
*BCORL1*	10 (1.2)	6 (1.1)	4 (1.3)	0.823
*FAT1*	10 (1.2)	7 (1.3)	3 (1.0)	0.660
*CUX1*	10 (1.2)	9 (1.7)	1 (0.3)	0.079

*MDS* myelodysplastic syndrome; *ANC* absolute neutrophil count; *BM* bone marrow; *WHO* World Health Organization (2016 classification); *SLD* single-lineage dysplasia; *MLD* multilineage dysplasia *RS*: ring sideroblasts; *EB* excess blasts; *IPSS*-*R* International Prognostic Scoring System-Revised; *IPSS*-*M* International Prognostic Scoring System-Molecular

*P* value: < 60 years vs. ≥ 60 years MDS patients

^*^Median (inter-quartile ranges)

**Fig. 5:**
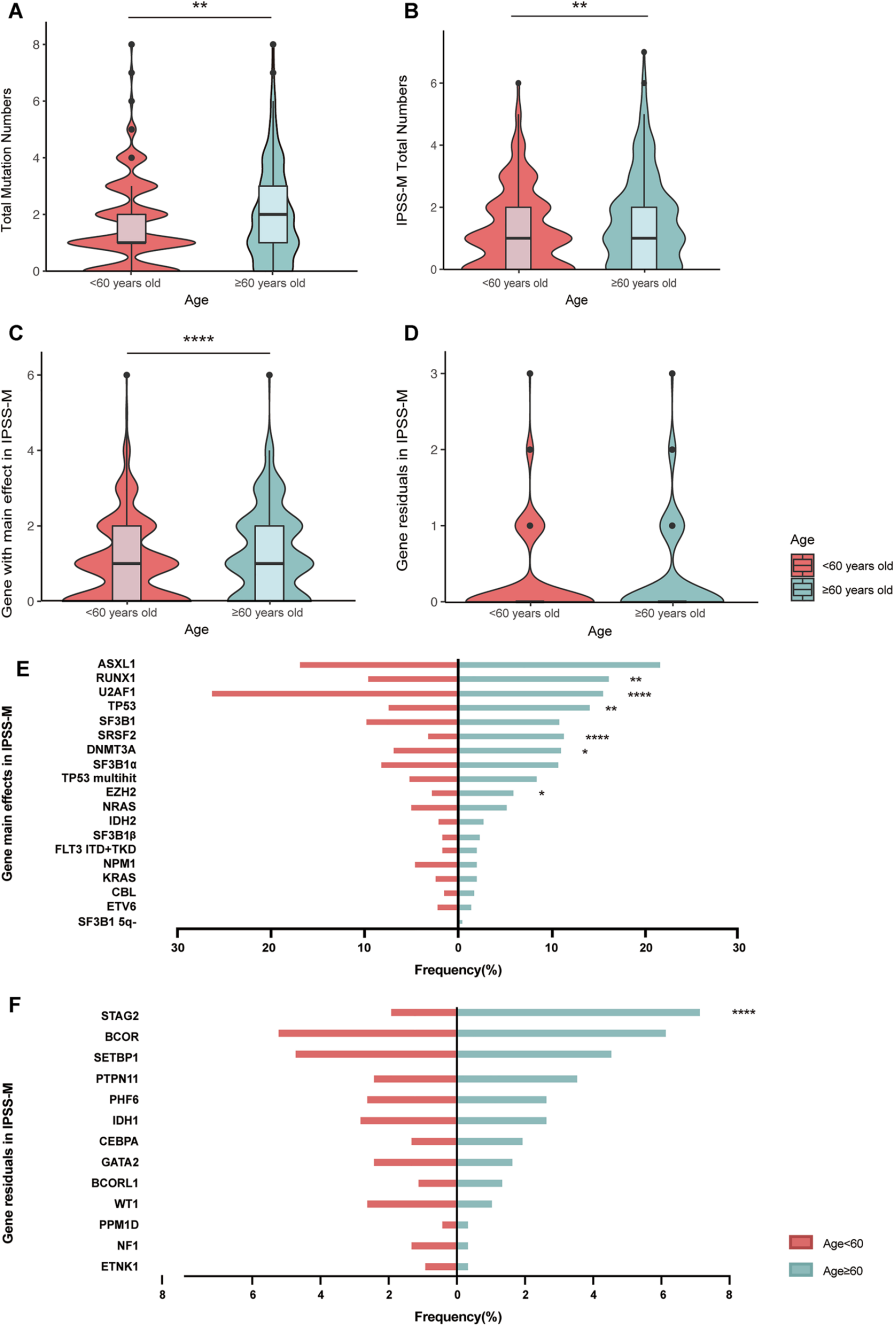
Mutational spectrum in the younger patients (age < 60 years) and older ones (age ≥ 60 years) with MDS. **A** The total number of mutations in the younger and older cohorts. **B**–**D** Genes related to IPSS-M in younger and older patients, including total number of genes (**B**), main effect genes (**C**) and residual genes in IPSS-M (**D**). **E**, **F** Prevalence of main effect genes and residual genes in IPSS-M of different age cohorts. 16 main effect genes in IPSS-M: *ASXL1, CBL, DNMT3A, ETV6, EZH2, FLT3, IDH2, KRAS, MLL*^*PTD*^*, NPM1, NRAS, RUNX1, SF3B1, SRSF2, TP53*^*multihit*^ and *U2AF1*; 15 residual genes in IPSS-M: *BCOR, BCORL1, CEBPA, ETNK1, GATA2, GNB1, IDH1, NF1, PHF6, PPM1D, PRPF8, PTPN11, SETBP1, STAG2* and *WT1*. Abbreviations: IPSS-M: International Prognostic Scoring System-Molecular

The frequency and distribution of mutations by age cohort is displayed in Fig. 5. Average number of mutations in subjects ≥ 60 years was greater compared with subjects < 60 years, 2.0 ± 1.7 *versus* 1.6 ± 1.5 (mean ± SD, *P* = 0.003). Mean number of IPSS-M mutated genes was also higher, 1.6 ± 1.4 *vs.* 1.3 ± 1.3 (*P* = 0.006) as was mean number of main effect genes, 1.3 ± 1.1 *versus* 1.0 ± 1.1 (*P* < 0.0001). There was no significant difference in IPSS-M residual genes (0.3 ± 0.6 *versus* 0.3 ± 0.6; *P* = 0.53, Fig. 5).

Figure 5 and Table 2 displays frequencies of mutated genes by age cohort. Subjects ≥ 60 years had a higher incidence of mutations in *RUNX1, TP53*, *TET2, SRSF2, DNMT3A, STAG2*, *EZH2* and *DDX41*. In contrast, mutations in *U2AF1* were more common in persons < 60 years (*P* < 0.05, Fig. 5, Table 2).

### Survival prediction accuracy of IPSS-R compared with IPSS-M by age.

The IPSS-R model had higher C- statistics (0.67 [95% CI 0.61, 0.72] *vs.* 0.65 [0.59, 0.71]) in younger cohort while IPSS-M had higher C- statistics (0.69 [0.64, 0.73] *vs.* 0.66 [0.61, 0.71]) in older cohort, suggesting that IPSS-M was more reliable than IPSS-R in patients aged 60 years or older (Fig. 4).

## Discussion

Incorporation of significant genetic mutations into conventional prognostic scoring tools undoubtedly provided more appropriate risk stratification of MDS patients [3–8]. However, there was a heterogeneity of patient populations, analysis methods and mutation inclusions in different models. Also, how to implement various genomic-clinical risk scoring systems in clinical practice still remained a challenge. In this study, we employed the IPSS-R and IPSS-M risk models in our cohort of 852 patients with de novo MDS to assess their prognostic strength.

Compared with IPSS-R categories, the utility of the IPSS-M model showed similar statistical value (C- statistics: 0.68 *vs.* 0.67) and the C- statistics was not as high as that of the IWG-PM cohort (C- statistics: 0.73) in Bernard et al. study [6].

Different disease proportions and patient populations may be part of the explanation. Only patients with de novo MDS were included in our study but approximately 20% of subjects in IPSS-M discovery cohort were diagnosed with secondary/therapy-related MDS (s/t-MDS) or MDS/myeloproliferative neoplasm overlap syndromes [6]. Examining baseline patient characteristics, the median age was much younger in our cohort compared with the IPSS-M cohorts (median age: 56 *vs.* 73 *vs.* 72 years, *P* < 0.001, Additional file 1: Table S1). An earlier onset age in Chinese MDS patients might be related to distinct genetic factors and different ethnic backgrounds [14–18]. Furthermore, our cohort showed pronounced cytopenia, increased BM blasts and unfavorable karyotypes compared to the IWG-PM cohort. Previous reports also indicated the phenomenon that Asian patients had more severe cytopenias and worse cytogenetic aberrations compared to Western patients [14–17]. Therefore, the ethnic specificities could also explain why IPSS-M did not show improved prognostic accuracy in our cohort.

In fact, MDS prognostic scoring systems were mainly established using clinical and genetic data obtained from patients aged 60 years or older. Although Kuendgen et al. [19] held the view that younger MDS patients were not significantly different compared with older ones. Other studies indicated that younger patients with MDS represented unique clinical and biological features and different prognosis [18, 20–22]. Therefore, we expanded our analyses to find whether the genomic-clinical risk model derived from older patients had different performance in different age cohorts.

Hence our patients were segregated into two cohorts using the cut-off age of 60 years depending on UN and WHO recommendations [23, 24]. The result highlighted that prognosis was more favorable in younger patients compared with older ones which is consistent with other reports [18, 19, 21]. The disparity in survival largely resulted from the elderly’s comorbidities.

Moreover, we found that differences arose from patients in younger and older cohorts. For patients belonging to the older cohort, they were more likely to be males which was in accordance with other investigations [18]. A similar proportion of MDS-EB cases in subjects < 60 years were found in prior single-center studies [19, 21, 22], which was markedly lower than that in older ones [18]. In parallel, the percentage of BM blasts was higher in subjects ≥ 60 years. Regarding cytogenetic aberrations, very poor IPSS-R cytogenetics were more frequent in the elderly. As is shown in our result, there is a trend towards disease progression for MDS subjects ≥ 60 years. Li et al. [18] suggested that a higher female-to-male ratio, increased trisomy 8, less advanced disease in patients younger than 60 years old may be due to a stronger self-immune surveillance reaction and a weaker T-cell surveillance, higher prevalence of BM blasts may result in more advanced disease and worse prognosis in older subjects, which revealed different pathogenesis. Interestingly, more subjects ≥ 60 years were divided into high-risk IPSS-M classifications whereas the distributions among IPSS-R risk groups were similar. This leaded to a more detailed investigation into the mutational profiles of different age groups.

Compared with younger patients, older patients had more mutations and more harmful genes according to IPSS-M model. There was a preponderance of *U2AF1* in younger ones while *ASXL1* was more frequent in subjects ≥ 60 years which was also reported by another study [25]. This may provide a possible explanation that the rate of *U2AF1* was 22% in our whole cohort, which was higher than other Western cohorts [6, 8]. Results for other mutations showed that an enrichment for *RUNX1*, *TP53*, *TET2*, *SRSF2*, *DNMT3A*, *STAG2*, *EZH2*, *DDX41* in the elderly. Such conclusion aligned with data from several important research that *DNMT3A*, *ASXL1* and *TET2* initiated clonal hematopoietic expansion and the mutation frequencies rose dramatically with the aging process [26–30]. Peterson ZD and colleagues [22] found *TP53* mutations were the most common mutations (21%) in patients 20–50 years old with MDS. But our result showed that the *TP53* mutations were enriched in subjects ≥ 60 years (14.1% *vs.* 7.6%) and there was no difference in the frequency of *TP53*^*multihit*^ in these two subgroups (8.3% *vs.* 5.0%, *P* = 0.052). We hypothesized the difference of these results may be related to the use of a different sequencing panel coupled with a limited cohort size. Mutations in *RUNX1*, *SRSF2* and *EZH2* predicted unfavorable prognosis and *STAG2* accelerates leukemogenesis process in MDS [8, 9, 31, 32]. Besides, germline *DDX41* mutations induce disease with an age ranging from 44–88 years and are associated with advanced disease, such as MDS-EB [33]. Although *DDX41* mutations occurred more frequently in older patients, germline and somatic *DDX41* mutations were not distinguished in our analysis. Taken together, the accumulation of detrimental mutations determined the clone evolution of MDS and was related to worse survival in the elderly. Furthermore, the different genetic profiles between Western MDS subjects and our subjects may result from different age distributions.

Next we tested survival prediction accuracy based on age < or ≥ 60 years. Remarkably, advanced improvement in the predictive power of IPSS-M was observed in patients ≥ 60 years old (C- statistics: 0.69 *vs.* 0.66), but it had not been found to be more predictive than IPSS-R in younger patients (C- statistics: 0.65 *vs.* 0.67).

However, our study had limitations. Our study was a retrospective analysis from a single center and 592 patients used a 141-gene panel without *PRPF8* and *GNB1* which were included in IPSS-M model. Nevertheless, we compared the patients’ characteristics to eliminate the influence. In general, a multicenter study is needed to confirm the prognostic value of IPSS-M and the heterogeneity of different age cohorts in MDS.

## Conclusions

In conclusion, the evidence that similar prognostic value between IPSS-R and IPSS-M prognostic models in our cohort could be explained by study-specific factors, like different population age, distinct disease proportions and unique ethnic backgrounds. Our study cohort was separated into younger and older cohorts because an earlier onset age was found. More advanced disease and increasing putative mutations were presented in patients aged 60 years or older. For them, IPSS-M model will undoubtedly enhance the ability of predicting prognosis and guiding proper therapy selections compared with IPSS-R model.

## Supplementary Information

**Additional file 1: Figure S1.** Re-stratification of patients from IPSS-R to IPSS-M. (A) Kaplan-Meier probability estimates of overall survival (OS) per IPSS-M category within each IPSS-R category. (B) Kaplan-Meier probability estimates of OS per IPSS-R category within each IPSS-M category.* P-values* are from the log-rank test. **Figure S2.** Overall survival of younger patients (age < 60 years) and older patients (age ≥ 60 years) with MDS stratified according to different prognostic scoring systems. (A) Kaplan–Meier curves of overall survival in the younger patients (age < 60 years) and older ones (age ≥ 60 years) with MDS. The younger patients showed significantly longer overall survival (OS) than the older ones (*P*<0.0001). (B-C) Kaplan-Meier representation of IPSS-R scoring system in younger and older patients. (D-E) Kaplan-Meier representation of IPSS-M scoring system in younger and older patients. *P-*values are from the log-rank test. **Table S1. **Clinical and laboratory characteristics of Chinese, Japan (validation cohort) and IWG-PM (discovery cohort) cohorts restricted to patients with primary MDS and untreated with disease-modifying therapies during their clinical course. **Table S2.** List of 141 genes included in the targeted sequencing panel. **Table S3.** List of 267 genes included in the targeted sequencing panel. **Table S4.** Clinical and laboratory characteristics of 592 and 260 patients in our cohort using different gene panels. **Table S5. **Clinical and laboratory characteristics of Chinese, Japan (validation cohort) and IWG-PM (discovery cohort) cohorts. **Table S6. **Distribution (%) of MDS patients categorized into IPSS-M categories by IPSS-R categories. **Table S7. **Distribution (%) of MDS patients categorized into IPSS-M categories by IPSS-R categories (by merging moderate low and moderate high into moderate in IPSS-M).

## Data Availability

The data that support the findings of this study are available on request from the corresponding author. The data are not publicly due to privacy or ethical restrictions. All authors disclose no conflict of interest.

## Abbreviations

MDS: Myelodysplastic syndromes
IPSS-R: The Revised International Prognostic Scoring System
IPSS-M: The molecular IPSS
WHO: The World Health Organization
IQR: Interquartile range
G-CSF: Granulocyte-colony stimulating factor
OS: Overall survival
AUROC: Area under receiver-operator characteristic
C-statistic: Concordance statistic
HR: Hazard Ratio
CI: Confidence Interval
MDS-EB: MDS with excess blasts
s/t-MDS: Secondary/therapy-related MDS
