# Supplementary material for: IPSS-M has greater survival predictive accuracy compared with IPSS-R in persons ≥ 60 years with myelodysplastic syndromes
Source: Exp Hematol Oncol. 2022 Oct 17;11:73. doi: 10.1186/s40164-022-00328-4 (PMC9578211; doi:10.1186/s40164-022-00328-4)
Supplement: Supplementary file 1 — Additional file 1: Figure S1. Re-stratification of patients from IPSS-R to IPSS-M. (A) Kaplan-Meier probability estimates of overall survival (OS) per IPSS-M category within each IPSS-R category. (B) Kaplan-Meier probability estimates of OS per IPSS-R category within each IPSS-M category. P-values are from the log-rank test. Figure S2. Overall survival of younger patients (age < 60 years) and older patients (age ≥ 60 years) with MDS stratified according to different prognostic scoring systems. (A) Kaplan–Meier curves of overall survival in the younger patients (age < 60 years) and older ones (age ≥ 60 years) with MDS. The younger patients showed significantly longer overall survival (OS) than the older ones (P<0.0001). (B-C) Kaplan-Meier representation of IPSS-R scoring system in younger and older patients. (D-E) Kaplan-Meier representation of IPSS-M scoring system in younger and older patients. P-values are from the log-rank test. Table S1. Clinical and laboratory characteristics of Chinese, Japan (validation cohort) and IWG-PM (discovery cohort) cohorts restricted to patients with primary MDS and untreated with disease-modifying therapies during their clinical course. Table S2. List of 141 genes included in the targeted sequencing panel. Table S3. List of 267 genes included in the targeted sequencing panel. Table S4. Clinical and laboratory characteristics of 592 and 260 patients in our cohort using different gene panels. Table S5. Clinical and laboratory characteristics of Chinese, Japan (validation cohort) and IWG-PM (discovery cohort) cohorts. Table S6. Distribution (%) of MDS patients categorized into IPSS-M categories by IPSS-R categories. Table S7. Distribution (%) of MDS patients categorized into IPSS-M categories by IPSS-R categories (by merging moderate low and moderate high into moderate in IPSS-M). [file 40164_2022_328_MOESM1_ESM.docx]

**Supplemental Material**

**Targeted gene sequencing:**

Targeted sequencing was performed on 141 genes across 592 MDS patients from August 2016 to March 2020 (Table S1). And a targeted gene sequencing of 267 known or putative genes were examined in the remaining 260 patients from April 2020 to September 2021 (Table S2). To eliminate the bias, we compared clinical data for the two subsets of patients (Table S3). Bi-allelic TP53 was determined as described^1, 2^: (1) 2 or more TP53 gene variants were detected, regardless of the VAF; (2) at least 1 TP53 gene variant co-occurred with a cytogenetic aberration involving chromosome 17p (eg, abnormality of 17p or monosomy 17); or (3) TP53 mutations were detected with a VAF > 55%. In addition to the putative oncogenic variants, we grouped mutations into 16 main effect genes (ASXL1, CBL, DNMT3A, ETV6, EZH2, FLT3, IDH2, KRAS, MLL^PTD^, NPM1, NRAS, RUNX1, SF3B1, SRSF2, TP53^multihit^ and U2AF1) and 15 residual genes (BCOR, BCORL1, CEBPA, ETNK1, GATA2, GNB1, IDH1, NF1, PHF6, PPM1D, PRPF8, PTPN11, SETBP1, STAG2 and WT1) depending on the IPSS-M model^3^. DNA from bone marrow were extracted and prepared for sequencing. Each DNA sample is quantiﬁed by gel electrophoresis and Nanodrop (Thermo). Libraries were prepared using Illumina standard protocol. The amplified DNA was captured with a 141 or a 267 Gene Panel using biotinylated oligo-probes (MyGenostics GenCap Enrichment technologies, Beijing, China). The capture experiment was conducted according to the manufacturer’s protocol^4^. Illumina utilizes a unique "bridged" amplification reaction that occurs on the surface of the flow cell. A flow cell containing millions of unique clusters is loaded into the HiSeq 2000 for automated cycles of extension and imaging. Illumina's Sequencing-by-Synthesis utilizes four proprietary nucleotides possessing reversible fluorophore and termination properties. Each sequencing cycle occurs in the presence of all four nucleotides leading to higher accuracy than methods where only one nucleotide is present in the reaction mix at a time. This cycle is repeated, one base at a time, generating a series of images each representing a single base extension at a specific cluster.

The average gene coverage was 98.1%. The average read depth was 1314×. Also, 95% of targeted regions were covered with greater than 20×.

**Basic bioinformatics analysis:**

For nuclear gene sequencing analysis, high-quality reads were retrieved from raw reads by filtering out the low quality reads and adaptor sequences using the Solexa QA package and the cutadapt program (<http://code.google.com/p/cutadapt/>), respectively. SOAPaligner program was then used to align the clean read sequences to the human reference genome (hg19).

After the PCR duplicates were removed by the Picard software, the SNPs was firstly identified using the SOAPsnp program (<http://soap.genomics.org.cn/soapsnp.html>). Subsequently, we realigned the reads to the reference genome using BWA and identified the insertions or deletions (InDels) using the GATK program (<http://www.broadinstitute.org/gsa/wiki/index.php/Home_Page>). The identified SNPs and InDels were annotated using the Exome-assistant program (<http://122.228.158.106/exomeassistant>). MagicViewer was used to view the short read alignment and validate the candidate SNPs and InDels.

**Sequence variant annotation:**

Once low confidence (Depth <5) or likely polymorphisms were removed from the dataset, each high confidence variant was filtered based on the information retrieved from public database(1000 Genomes, ESP6500, Inhouse, PolyPhen, SIFT and COSMIC). The remaining variants were considered as candidate somatic mutations, and were finally tagged as oncogenic and possible oncogenic association with myeloid disease based on the information derived from the literature, the Catalog of Somatic Mutations in Cancer(COSMIC).

a. Oncogenic

• Known myeloid malignancies related oncogenic variants previously reported in the literature;

• Truncating variants (nonsense mutations, essential splice mutations or frameshift indels) in genes implicated in myeloid malignancies through acquisition of loss of function mutations.

b. Possible oncogenic

• Previously unreported variants that cluster (±3aa) with known myeloid malignancies related oncogenic variants in COSMIC.

**Statistical methods:**

All data were analysed using SPSS (version 25.0) and R statistical language. Numerical variables were described by median and inter-quartile ranges, while categorical variables were summarized with count and relative frequency. Categorical variables were evaluated by the Fisher exact test or the χ2 test and continuous variables were calculated by the Mann-Whitney U test or Kruskal-Wallis analysis. Overall survival (OS) was defined as the time from diagnosis to death or last follow-up and univariate analyses of OS were calculated by the Kaplan-Meier method and compared by the log-rank test. Multivariate survival analyses were performed in patients by applying Cox proportional-hazards regression model which included significant variables in the univariate analyses (*P-value* <0.2). The statistical predictive power of the prognostic soring models was assessed by the time-depend area under receiver-operator characteristic (AUROC) curves expressed as a Concordance (C)-statistic^5^, a higher C-statistic indicates better quality of classification. Data were analysed using SPSS version 25.0 (IBM, Chicago, IL, USA) and R statistical language (R Development Core 2008).

**References**

1. Bernard E, Nannya Y, Hasserjian RP, Devlin SM, Tuechler H, Medina-Martinez JS, et al. Implications of TP53 allelic state for genome stability, clinical presentation and outcomes in myelodysplastic syndromes. Nature medicine. 2020;26(10):1549-56.

2. Grob T, Al Hinai ASA, Sanders MA, Kavelaars FG, Rijken M, Gradowska PL, et al. Molecular characterization of mutant TP53 acute myeloid leukemia and high-risk myelodysplastic syndrome. Blood. 2022;139(15):2347-54.

3. Bernard E, Tuechler H, Greenberg PL, Hasserjian RP, Arango Ossa JE, Nannya Y, et al. Molecular International Prognostic Scoring System for Myelodysplastic Syndromes. NEJM Evidence. 2022;1(7).

4. Li B, Gale RP, Xu Z, Qin T, Song Z, Zhang P, et al. Non-driver mutations in myeloproliferative neoplasm-associated myelofibrosis. J Hematol Oncol. 2017;10(1):99.

5. Harrell FE. Evaluating the Yield of Medical Tests. JAMA: The Journal of the American Medical Association. 1982;247(18).

**Figure S1. Re-stratification of patients from IPSS-R to IPSS-M.** (A) Kaplan-Meier probability estimates of overall survival (OS) per IPSS-M category within each IPSS-R category. (B) Kaplan-Meier probability estimates of OS per IPSS-R category within each IPSS-M category. *P-values* are from the log-rank test.

Abbreviations: IPSS-R: International Prognostic Scoring System-Revised; IPSS-M: International Prognostic Scoring System-Molecular; OS: overall survival.

**
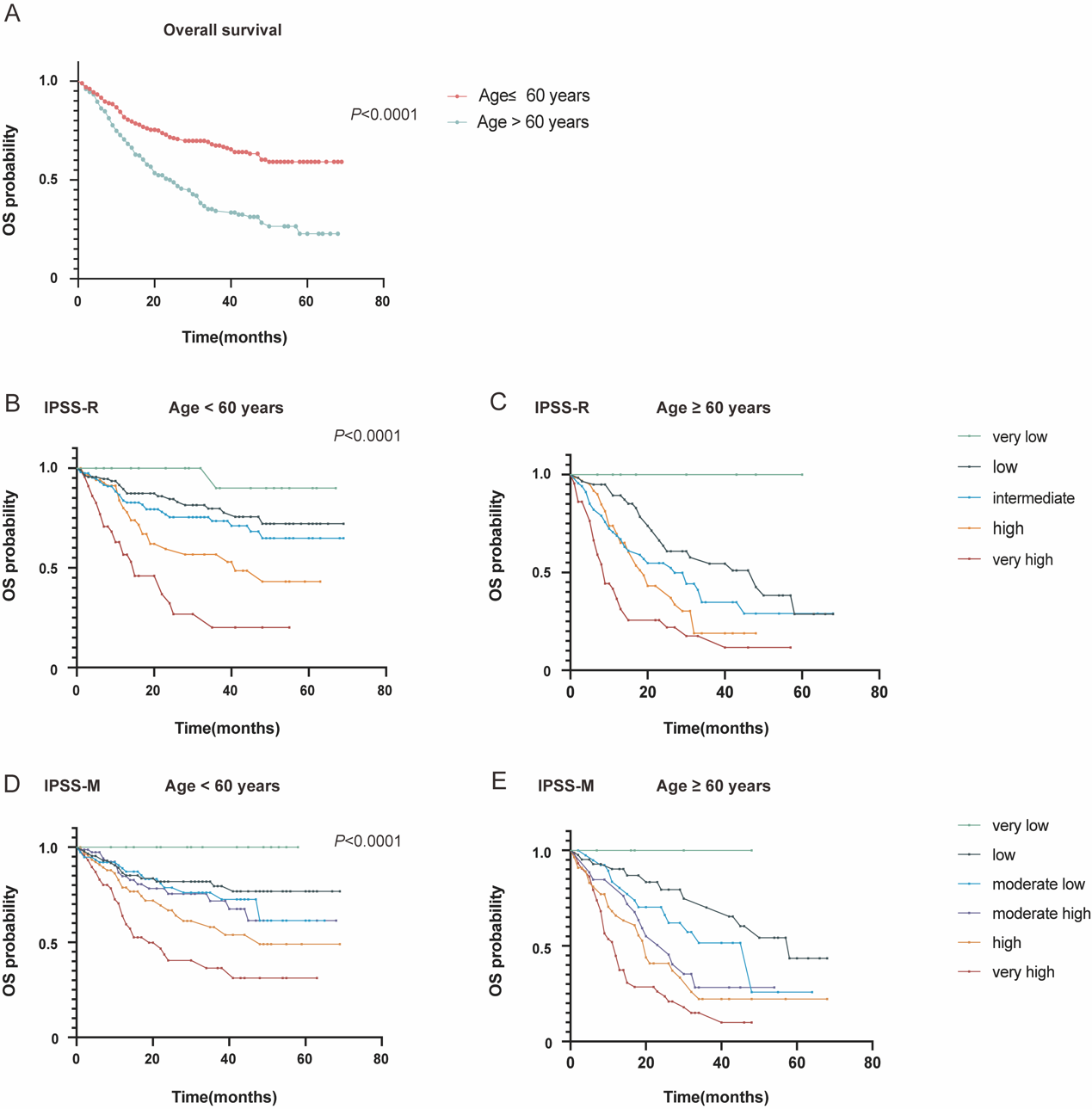
**

**Figure S2. Overall survival of younger patients (age < 60 years) and older patients (age ≥ 60 years) with MDS stratified according to different prognostic scoring systems.** (A) Kaplan–Meier curves of overall survival in the younger patients (age < 60 years) and older ones (age ≥ 60 years) with MDS. The younger patients showed significantly longer overall survival (OS) than the older ones (*P*<0.0001). (B-C) Kaplan-Meier representation of IPSS-R scoring system in younger and older patients. (D-E) Kaplan-Meier representation of IPSS-M scoring system in younger and older patients. *P-*values are from the log-rank test.

Abbreviations: IPSS-R: International Prognostic Scoring System-Revised; IPSS-M: International Prognostic Scoring System-Molecular; OS: overall survival. **Table S1.** Clinical and laboratory characteristics of Chinese, Japan (validation cohort) and IWG-PM (discovery cohort) cohorts restricted to patients with primary MDS and untreated with disease-modifying therapies during their clinical course.

| Characteristic | Chinese cohort（n=852） | Japan cohort（n=522) | IWG-PM（n=1879） | *P1* | *P2* | *P3* |
| --- | --- | --- | --- | --- | --- | --- |
| Age (years) * | 56（44～64） | 72（66～79） | 73（65～80） | <0.001 | <0.001 | 0.15 |
| Sex n (%) |  |  |  | 0.077 | 0.033 | <0.001 |
| Male | 550（64.6） | 362（69） | 1131（60） |  |  |  |
| Female | 302（35.4） | 160（31） | 748（40） |  |  |  |
| BM blasts (%) * | 2.5（1～7） | 3（1～8） | 2（1～4） | 0.002 | 0.162 | <0.001 |
| (Missing) | 1 | 0 | 0 |  |  |  |
| Hemoglobin (g/L) * | 79（66～95） | 90（80～100） | 100（90～110） | <0.001 | <0.001 | <0.001 |
| (Missing) | 0 | 0 | 74 |  |  |  |
| Platelets (×10^9^/L) * | 60（31～119） | 82（42～150） | 148（79～251） | <0.001 | <0.001 | ＜0.001 |
| (Missing) | 0 | 0 | 86 |  |  |  |
| ANC (×10^9^/L) * | 1（0.7～2） | 1（1～2） | 2（1～4） | 0.02 | <0.001 | ＜0.001 |
| (Missing) | 0 | 13 | 126 |  |  |  |
| IPSS-R karyotype |  |  |  | ＜0.001 | <0.001 | ＜0.001 |
| Very good | 10（1.2） | 11（2.1） | 108（6.7） |  |  |  |
| Good | 427（50.1） | 258（49） | 1179（73） |  |  |  |
| Intermediate | 186（21.8） | 101（19） | 215（13） |  |  |  |
| Poor | 42（4.9） | 52（10） | 39（2.4） |  |  |  |
| Very poor | 95（11.2） | 100（19） | 69（4.3） |  |  |  |
| (Missing) | 92 | 0 | 269 |  |  |  |
| IPSS-R Prognostic Risk Score |  |  |  | ＜0.001 | <0.001 | ＜0.001 |
| Very low | 28（3.3） | 42（8.3） | 418（24） |  |  |  |
| Low | 185（21.7） | 135（27） | 788（45） |  |  |  |
| Intermediate | 241（28.3） | 115（23） | 323（19） |  |  |  |
| High | 175（20.5） | 77（15） | 126（7.2） |  |  |  |
| Very high | 131（15.4） | 140（28） | 88（5.0） |  |  |  |
| (Missing) | 92 | 13 | 136 |  |  |  |
| WHO 2016 subtypes |  |  |  |  |  |  |
| MDS-SLD/MLD | 414（48.6） | 189（36） | 692（38） |  |  |  |
| MDS-RS-SLD/MLD | 46（5.4） | 32（6.1） | 357（20） |  |  |  |
| MDS-EB1/2 | 359（42.1） | 258（49） | 360（20） |  |  |  |
| 5q- syndrome | 12（1.4） | 7（1.3） | 75（4.1） |  |  |  |
| Unclassified MDS | 21（2.5） | 16（3.1） | 56（3.1） |  |  |  |
| CMML | 0（0） | 11（2.1） | 199（11） |  |  |  |
| MDS/MPN-RS-T | 0（0） | 3（0.6） | 35（1.9） |  |  |  |
| MDS/MPN-U | 0（0） | 10（1.3） | 34（1.9） |  |  |  |
| other | 0（0） | 6（1.1） | 7（0.4） |  |  |  |
| (Missing) | 92 | 0 | 64 |  |  |  |

Abbreviations: MDS: myelodysplastic syndrome; IWG-PM: International Working Group for Prognosis in MDS; BM: bone marrow; ANC: absolute neutrophil count; IPSS-R: International Prognostic Scoring System-Revised; WHO: World Health Organization; SLD: single-lineage dysplasia; MLD: multilineage dysplasia; RS: ring sideroblasts; EB: excess blasts; CMML: chronic myelomonocytic leukemia; MPN: myeloproliferative neoplasm; MDS/MPN-RS-T: MDS/MPN with ring sideroblasts and thrombocytosis; MDS/MPN-U: MDS/MPN-unclassifiable.

*P* value: *P1*: Chinese cohort *vs.* Japan cohort; *P2*: Chinese cohort *vs.* IWG-PM; *P3*: Japan cohort *vs.* IWG-PM.

*: Median (inter-quartile ranges).

**Table S2.** List of 141 genes included in the targeted sequencing panel.

| ABL1 | ANKRD26 | ARID1A | ASXL1 | ASXL2 | ATG2B | ATM | B2M | BCL2 | BCL6 |
| --- | --- | --- | --- | --- | --- | --- | --- | --- | --- |
| BCOR | BCORL1 | BIRC3 | BRAF | BRINP3 | BTK | CALR | CARD11 | CASP8 | CBL |
| CCND1 | CCND2 | CCND3 | CCR4 | CD28 | CD58 | CD798 | CDC25C | CDKN1B | CDKN2A |
| CEBPA | CNOT3 | CREBBP | CRLF2 | CSF3R | CSNK1A1 | CUX1 | CXCR4 | DDX3X | DDX41 |
| DIS3 | DNM2 | DNMT3A | DNMT3B | EED | EGR1 | EP300 | ETNK1 | ETV6 | EZH2 |
| FAM46C | FAT1 | FBXW7 | FGFR3 | FLT3 | GATA1 | GATA2 | GATA3 | GNA13 | ID3 |
| IDH1 | IDH2 | IKZF1 | IL7R | IRF4 | JAK1 | JAK2 | JAK3 | KDM6A | KIT |
| KLF2 | KMT2A | KMT2D | KRAS | MAP2K1 | MAPK1 | MAX | MED12 | MEF2B | MPL |
| MYC | MYD88 | NF1 | NOTCH1 | NOTCH2 | NPM1 | NRAS | NT5C2 | PAX5 | PDGFRB |
| PHF6 | PIGA | PLCG1 | PLCG2 | PPM1D | PRDM1 | PRKCB | PRPS1 | PTEN | PTPN11 |
| RAD21 | RBBP6 | RELN | RHOA | RPL10 | RUNX1 | SETBP1 | SETD2 | SF1 | SF3B1 |
| SH2B3 | SMC1A | SMC3 | SPEN | SRP72 | SRSF2 | STAG2 | STAT3 | STAT5B | SUZ12 |
| TAL1 | TCF3 | TERT | TET2 | TNFAIP3 | TNFRSF14 | TP53 | TPMT | TRAF3 | U2AF1 |
| USP7 | WHSC1 | WT1 | XPO1 | ZBTB7A | ZMYM3 | ZRSR2 | NOTCH3 | NOTCH4 | PRPF8 |
| ZNF384 |  |  |  |  |  |  |  |  |  |

**Table S3.** List of 267 genes included in the targeted sequencing panel.

| ABCB1 | ABL1 | ANKRD26 | APC | ARID1A | ARID1B | ARID2 | ARID5B | ASXL1 | ASXL2 |
| --- | --- | --- | --- | --- | --- | --- | --- | --- | --- |
| ATG2B | ATM | ATRX | B2M | BACH2 | BCL10 | BCL2 | BCL6 | BCL7A | BCOR |
| BCORL1 | BIRC3 | BLM | BPGM | BRAF | BRCA1 | BRCA2 | BRIP1 | BTG1 | BTG2 |
| BTK | CALR | CARD11 | CBL | CBLB | BCLC | CCND1 | CCND3 | CCR4 | CD28 |
| CD58 | CD79A | CD79B | CDC25C | CDKN1A | CDKN1B | CDKN2A | CDKN2B | CDKN2C | CEBPA |
| CHD2 | CHD8 | CIITA | CNOT3 | CREBBP | CRLF2 | CSF1R | CSF3R | CSMD1 | CSNK1A1 |
| CTCF | CUX1 | CXCR4 | CYLD | DDX3X | DDX41 | DIS3 | DKC1 | DNM2 | DNMT3A |
| DNMT3B | DTX1 | DUSP2 | EBF1 | EED | EGFR | EGLN1 | EGR1 | ELANE | EP300 |
| EPHA7 | EPOR | ETNK1 | ETV6 | EZH2 | FAM46C | FAS | FAT1 | FAT4 | FBXO11 |
| FBXW7 | FGFR3 | FLT3 | FOXO1 | FYN | GAB2 | GATA1 | GATA2 | GATA3 | GFI1 |
| GNA13 | GNAI2 | GNAS | GNB1 | GSKIP | H1-2 | H1-3 | H1-4 | H1-5 | HAX1 |
| HLA-A | HLA-C | HLA-DMB | HNRNPK | HRAS | HUWEI | HVCN1 | ID3 | IDH1 | IDH2 |
| IGLL5 | IKZF1 | IKZF2 | IKZF3 | IL7R | IRF2BP2 | IRF4 | IRF8 | ITPKB | JAK1 |
| JAK2 | JAK3 | JUNB | KDM6A | KIT | KLF2 | KLHL6 | KMT2A | KMT2B | KMT2C |
| KMT2D | KRAS | KRT20 | LCOR | LMO2 | LTB | LYN | MAP2K1 | MAPK1 | MAX |
| MCL1 | MED12 | MEF2B | MFHAS1 | MPL | MTOR | MYC | MYCN | MYD88 | MYOM2 |
| NF1 | NFE2 | NFKBIA | NFKBIE | NOTCH1 | NOTCH2 | NOTCH3 | NOTCH4 | NPM1 | NRAS |
| NT5C2 | P2RY8 | PALB2 | PAX5 | PDGFRA | PDGFRB | PDS5B | PHF6 | PIGA | PIK3CA |
| PIK3CD | PIK3R1 | PIM1 | PIM2 | PLCG1 | PLCG2 | POT1 | PPM1D | PRDM1 | PRF1 |
| PRKCB | PRKD2 | PRKDC | PRPF8 | PRPS1 | PSMB5 | PTEN | PTPN1 | PTPN11 | PTPRD |
| RAD12 | RASA2 | RB1 | RBBP6 | RELN | RHOA | RPL10 | RRAGC | RUNX1 | SAMHD1 |
| SBDS | SETBP1 | SETD1B | SETD2 | SETDB1 | SF1 | SF3B1 | SGK1 | SH2B3 | SH2D1A |
| SMARCA4 | SMC1A | SMC3 | SMO | SOCS1 | SP140 | SPEN | SRP72 | SRSF2 | STAG2 |
| STAT3 | STAT5B | STAT6 | SUFU | SUZ12 | SYK | TAL1 | TBL1XR1 | TCF3 | TERC |
| TERT | TET1 | TET2 | TMEM30A | TMSB4X | TNFAIP3 | TNFRSF14 | TOX | TP53 | TPMT |
| TRAF3 | U2AF1 | UBE2A | UBR5 | USP7 | VAV1 | VHL | WHSC1 | WT1 | XBP1 |
| XPO1 | ZAP70 | ZBTB7A | ZFP36L1 | ZMYM3 | ZNF292 | ZRSR2 |  |  |  |

**Table S4.** Clinical and laboratory characteristics of 592 and 260 patients in our cohort using different gene panels.

| Characteristic | 141-gene panel（n=592） | 267-gene panel（n=260) | *P-*value |
| --- | --- | --- | --- |
| Age (years) * | 55（43～63） | 57（46～64） | 0.153 |
| Sex n (%) |  |  | 0.422 |
| Male | 377（63.7） | 173（66.5） |  |
| Female | 215（36.3） | 87（28.8） |  |
| BM blasts (%) * | 2.5（1.0～6.5） | 3（1～7） | 0.232 |
| (Missing) | 0 | 1 |  |
| Hemoglobin (g/L) * | 79（67～96） | 78（63～95） | 0.277 |
| (Missing) | 0 | 0 |  |
| Platelets (×10^9^/L) * | 59（32～115） | 61（28～126） | 0.833 |
| (Missing) | 0 | 0 |  |
| ANC (×10^9^/L) * | 1.1（0.7～2） | 1.1（0.6～2） | 0.746 |
| (Missing) | 0 | 0 |  |
| IPSS-R karyotype |  |  | 0.101 |
| Very good | 5（1.0） | 5（2.1） |  |
| Good | 302（58.2） | 125（51.9） |  |
| Intermediate | 114（22.0） | 72（29.9） |  |
| Poor | 29（5.6） | 13（5.4） |  |
| Very poor | 69（13.3） | 26（10.8） |  |
| (Missing) | 73 | 19 |  |
| IPSS-R category |  |  | 0.879 |
| Very low | 20（3.9） | 8（3.3） |  |
| Low | 129（24.9） | 56（23.2） |  |
| Intermediate | 165（31.8） | 76（31.5） |  |
| High | 114（22.0） | 61（25.3） |  |
| Very high | 91（17.5） | 40（16.6） |  |
| (Missing) | 73 | 19 |  |
| IPSS-M category |  |  | 0.395 |
| Very low | 13（2.5） | 8（3.3） |  |
| Low | 99（19.1） | 39（16.2） |  |
| Moderate low | 89（17.2） | 36（14.9） |  |
| Moderate high | 82（15.8） | 31（12.9） |  |
| High | 121（23.4） | 56（23.2） |  |
| Very high | 14（2.5） | 71（29.5） |  |
| (Missing) | 73 | 19 |  |
| WHO 2016 subtypes |  |  | 0.054 |
| MDS-SLD/MLD | 304（51.3） | 110（42.3） |  |
| MDS-RS-SLD/MLD | 33（5.6） | 13（5.0） |  |
| MDS-EB1/2 | 233（39.4） | 126（48.5） |  |
| 5q- syndrome | 6（1.0） | 6（2.3） |  |
| Unclassified MDS | 16（2.7） | 5（1.9） |  |
| (Missing) | 0 | 0 |  |

Abbreviations: MDS: myelodysplastic syndrome; ANC: absolute neutrophil count; BM: bone marrow; WHO: World Health Organization (2016 classification); SLD: single-lineage dysplasia; MLD: multilineage dysplasia; RS: ring sideroblasts; EB: excess blasts; IPSS-R: International Prognostic Scoring System-Revised; IPSS-M: International Prognostic Scoring System–Molecular.

*P* value: ≤60 years vs. >60 years MDS patients.

*: Median (inter-quartile ranges).

**Table S5.** Clinical and laboratory characteristics of Chinese, Japan (validation cohort) and IWG-PM (discovery cohort) cohorts.

| Characteristic | Chinese cohort（n=852） | Japan cohort（n=754) | IWG-PM（n=2957） |
| --- | --- | --- | --- |
| Age (years) * | 56（44～64） | 71（64～78） | 72（63～78） |
| (Missing) | 0 | 21 | 20 |
| Sex n (%) |  |  |  |
| Male | 550（64.6） | 539（71） | 1776（60） |
| Female | 302（35.4） | 215（29） | 1181（40） |
| BM blasts (%) * | 2.5（1～7） | 5（1～10） | 3（1～6） |
| (Missing) | 1 | 0 | 104 |
| Hemoglobin (g/L) * | 79（66～95） | 80（70～100） | 100（80～110） |
| (Missing) | 0 | 0 | 105 |
| Platelets (×10^9^/L) * | 60（31～119） | 81（41～146） | 130（69～236） |
| (Missing) | 0 | 0 | 118 |
| ANC (×10^9^/L) * | 1（0.7～2） | 1（1～2） | 2（1～3） |
| (Missing) | 0 | 18 | 179 |
| IPSS-R karyotype |  |  |  |
| Very good | 10（1.2） | 16（2.1） | 119（4.6） |
| Good | 427（50.1） | 349（46） | 1786（69） |
| Intermediate | 186（21.8） | 139（18） | 356（14） |
| Poor | 42（4.9） | 71（9.4） | 121（4.7） |
| Very poor | 95（11.2） | 179（24） | 206（8.0） |
| (Missing) | 92 | 0 | 369 |
| IPSS-R Prognostic Risk Score |  |  |  |
| Very low | 28（3.3） | 48（6.5） | 489（18） |
| Low | 185（21.7） | 161（22） | 1078（39） |
| Intermediate | 241（28.3） | 160（22） | 562（20） |
| High | 175（20.5） | 144（20） | 355（13） |
| Very high | 131（15.4） | 223（30） | 269（9.8） |
| (Missing) | 92 | 18 | 204 |
| WHO 2016 subtypes |  |  |  |
| MDS-SLD/MLD | 407（47.8） | 246（33） | 921（32） |
| MDS-RS-SLD/MLD | 54（6.3） | 37（4.9） | 461（16） |
| MDS-EB1/2 | 358（42.0） | 415（55） | 887（31） |
| 5q- syndrome | 12（1.4） | 9（1.2） | 142（4.9） |
| Unclassified MDS | 21（2.5） | 19（2.5） | 85（3.0） |
| CMML | 0（0） | 14（1.9） | 272（9.5） |
| MDS/MPN-RS-T | 0（0） | 4（0.5） | 42（1.5） |
| MDS/MPN-U | 0（0） | 10（1.3） | 51（1.8） |
| other | 0（0） | 0（0） | 10（0.3） |
| (Missing) | 92 | 0 | 86 |
| Type |  |  |  |
| primary | 852（100） | 708（94） | 2641（92） |
| secondary/therapy-related | 0（0） | 46（6.1） | 234（8.1） |
| (Missing) | 0 | 0 | 82 |

Abbreviations: MDS: myelodysplastic syndrome; IWG-PM: International Working Group for Prognosis in MDS; BM: bone marrow; ANC: absolute neutrophil count; IPSS-R: International Prognostic Scoring System-Revised; WHO: World Health Organization; SLD: single-lineage dysplasia; MLD: multilineage dysplasia; RS: ring sideroblasts; EB: excess blasts; CMML: chronic myelomonocytic leukemia; MPN: myeloproliferative neoplasm; MDS/MPN-RS-T: MDS/MPN with ring sideroblasts and thrombocytosis; MDS/MPN-U: MDS/MPN-unclassifiable.

*: Median (inter-quartile ranges).

**Table S6.** Distribution (%) of MDS patients categorized into IPSS-M categories by IPSS-R categories.

|  |  | IPSS-M | | | | | | |
| --- | --- | --- | --- | --- | --- | --- | --- | --- |
| IPSS-R |  | Very low | Low | Moderate low | Moderate High | High | Very High | Total |
|  | Very low | 11 (39.3) | 16(57.1) | 1(3.6) | 0 | 0 | 0 | 28 |
|  | Low | 9 (4.9) | 93 (50.3) | 51 (27.6) | 20(10.8) | 7(3.8) | 5(2.7) | 185 |
|  | Intermediate | 0 | 28 (11.6) | 63 (26.1) | 67 (27.8) | 60(24.9) | 23(9.5) | 241 |
|  | High | 1(0.6) | 1(0.6) | 10(5.7) | 25 (14.3) | 74 (42.3) | 64(36.6) | 175 |
|  | Very high | 0 | 0 | 0 | 1 (0.8) | 29(22.3) | 101(76.9) | 131 |
|  | Total | 21 | 138 | 125 | 113 | 170 | 193 | 760 |

Abbreviations: MDS: myelodysplastic syndrome; IPSS-M: International Prognostic Scoring System–Molecular; IPSS-R: International Prognostic Scoring System-Revised.

**Table S7.** Distribution (%) of MDS patients categorized into IPSS-M categories by IPSS-R categories (by merging moderate low and moderate high into moderate in IPSS-M).

|  |  | IPSS-M | | | | | |
| --- | --- | --- | --- | --- | --- | --- | --- |
| IPSS-R |  | Very low | Low | Moderate | High | Very High | Total |
|  | Very low | 11 (39.3) | 16(57.1) | 1(3.6) | 0 | 0 | 28 |
|  | Low | 9 (4.9) | 93 (50.3) | 71 (38.4) | 7(3.8) | 5(2.7) | 185 |
|  | Intermediate | 0 | 28 (11.6) | 130 (53.9) | 60(24.9) | 23(9.5) | 241 |
|  | High | 1(0.6) | 1(0.6) | 35(20.0) | 74 (42.3) | 64(36.6) | 175 |
|  | Very high | 0 | 0 | 1(0.8) | 29(22.3) | 101(76.9) | 131 |
|  | Total | 21 | 138 | 238 | 170 | 193 | 760 |

Abbreviations: MDS, myelodysplastic syndrome; IPSS-M: International Prognostic Scoring System–Molecular; IPSS-R: International Prognostic Scoring System-Revised.
